# Physicians’ Accounts

**Published:** 1898-08

**Authors:** 


### PHYSICIANS' ACCOUNTS.

It is said, and certainly with a good deal of truth, that physicians generally make a living, but that few of them ever lay up much of this world's goods. The cause of this is twofold: first, because the physician is notoriously a poor business man; and, secondly, because the profession is so overcrowded that no single physician is apt to have a monopoly of professional work. Many a poor physician would be well off if he could only collect the honest bills that were due him. But, for some unknown reason, posterity has decreed that the physician's bill shall be the last to be paid. This we think is largely the result of the methods usually employed by physicians. "The laborer is worthy of his hire," and the physician should send his bill just as soon as his work is completed or the case dismissed. "Time softens many a wounded feeling" is true, but it also softens the spirit of payment after the patient has recovered. Dr. H. C. Wood's remarks are certainly apropos:

In the *American Medico-Surgical Bulletin* for June 25, 1898, Dr. H. C. Wood thus discourses concerning "Physicians' Accounts":

"If it be desired by any doctor to have his accounts kept in such a way that they shall bring a smile of approval to the stern judicial countenance, he should keep a day-book, in which should be entered the particulars of the visit, when it was made, how long it continued, what was done and medicines were given, the exact value according to the system of charging adopted of such medicine, the charging together in a single sum for 'professional services and medicines' being a 'lumping' of charges sufficiently irritating to the judicial mind to produce apoplexy in a testy judge, and sure to be thrown out. Probably few successful, and therefore busy and overworked physicians, would be willing to become a slave of the day-book, and to try in the wee hours of the morning to satisfy the judicial interrogators. Certainly the writer of the present article would rather be thought a fraud by the orphans' court judges and lose various bills than spend such a life of toil at the galley-oar of accounts.

"The case of the surgeon is still more difficult. The details of his work are so great and elaborate that the amount of bookkeeping required, according to the advice which we have heard given by lawyers of repute, would be overwhelming. It is asserted that the surgical book of original entry, to be satisfactory, should state how many visits were paid before and after the operation, and should also show exactly what was done and the time consumed in such visits, also the details of the operations, how long it took for etherization and other preparation, what amount of ether and other materials were used and the value of such ether and other materials, what was done at each stage of the operation in detail, and a history of the final after-treatment; also the number of assistants employed, and exactly what each assistant did. In fact, if it took an hour for the operation, it would probably take an hour and a half for its entrance in the book of entry.

"Under all the circumstances, it appears rather strange that the physicians are paid as well as they are, not that they lose so many accounts. The proper measure for decreasing the percentage of losses seems to us to lie not so much in proper bookkeeping as in the approximation to a cash basis. . . .

"All this is sufficient to make an old-fashioned physician shudder. We shall never forget the hours of unrest and anger produced in one of the old-time doctors by a patient having personally asked him for a bill instead of writing a note. 'Just,' as the old gentleman said, 'as if I were a carpenter or a mason.' Trade methods in their best aspects are, however, after all only proper methods of dealing between man and man; and if the profession as a profession desires to collect its just pecuniary dues, such trade methods must be to a greater or less extent adopted. Long credits ought to be abolished. Recently an account was rendered in our orphans' court for services rendered during a period of five years, and was very properly thrown out except for the last year. The possibility of fraud in such an account is, of course, very obvious: the natural supposition is, that the physician did not send his bill because he was afraid he would not get it from the patient while alive. In our opinion, the law should not allow a demand for payment of any professional service to be made more than a year after they are given. The right procedure for a doctor is to render his account at least every six months, and better every three months. A few of our leading and progressive surgeons in Philadelphia are now having their bills sent in monthly, with, as they assert, a very good effect upon their incomes."

